# Measurement properties of patient-reported outcome measures of mental help-seeking attitude: a systematic review of psychometric properties

**DOI:** 10.3389/fpsyt.2023.1182670

**Published:** 2023-07-19

**Authors:** Cheng Bian, Shu-Yan Chen, Shi-Rui Yan, Wei-Wei Zhao, Ru-Xuan Wang, Yin Cheng, Yan-Hong Zhang

**Affiliations:** ^1^School of Nursing, Nanjing Medical University, Nanjing, China; ^2^The Affiliated Brain Hospital of Nanjing Medical University, Nanjing, China

**Keywords:** help-seeking attitudes, mental disorders, measurement properties, systematic review, patient-reported outcome measures

## Abstract

**Background:**

At present, the phenomenon of patients with mental disorders not seeking mental help is very serious, and the mental help-seeking attitude is the central structure of the help process. However, there is no consensus on which patient-reported outcome measure (PROM) should be used to assess mental help-seeking attitudes.

**Objective:**

The systematic review aims to critically appraise, compare, and summarize the measurement quality of the all-available PROMs about mental help-seeking attitudes to provide evidence-based guidance and reference for clinical researchers.

**Methods:**

A systematic search was conducted in 9 databases (PubMed, Embase, Web of Science, Medline, APA, CINAHL, Sinomed, CNKI, and WanFang) since the establishment of the database until November 30, 2022 to identify articles on the PROMs of mental help-seeking attitudes. We used the COSMIN guidelines to evaluate the methodological quality and measurement properties of all-available PROMs, and a modified Grading, Recommendation, Assessment, Development, and Evaluation (GRADE) system to evaluate the level of evidence supporting each rating. Finally, the recommendation level is given according to the overall quality of each PROM.

**Results:**

We identified 29 studies representing 13 PROMs out of 2,828 screening studies. The overall quality of the included PROMs varied, with 6 rated as class B, 6 as class C, and only the Mental Help Seeking Attitudes Scale (MHSAS) as class A.

**Conclusion:**

The measurement characteristics of MHSAS have been the most comprehensive evaluation, and it has good reliability and validity, and high feasibility for clinical application, so it can be temporarily recommended for use, but the above conclusions still need to be supported by more high-quality evidence.

## 1. Introduction

In recent years, under the influence of population, environment, and social politics, mental health disorders have become a severe and costly global public health concern, affecting people of different ages, cultures, and socio-economic statuses (1, 2). At the same time, although the quality and effectiveness of mental health treatments and services have greatly improved, the utilization of mental health services is low (3, 4), resulting in a treatment gap that is wider than that in any other health sector (5). Results from a large-scale epidemiological study (6) show that fewer than one in five respondents with psychiatric disorders sought mental health services during the year they were interviewed. Many people who might benefit from psychotherapy are often reluctant to seek psychological help. A study (7) during the COVID-19 pandemic also validated the results. Mental disorders are treatable and possibly preventable, but the fact is that mental help-seeking is often delayed or completely absent (8–10). Some studies (11–13) have shown that the delay or refusal of psychological help will not only aggravate the negative experience of patients with mental illness, but will further increase the burden and cost, and eventually lead to severe ramifications for the individual. To close this treatment gap in mental health services, it is important to understand the influencing factors of the mental help-seeking process.

Mental help-seeking refers to the process of individuals seeking help from professionals when they encounter psychological troubles or obstacles. As one of the crucial variables affecting mental help-seeking (14), the attitudes towards mental help-seeking are people’s overall evaluation (i.e., good vs. bad) of the act of seeking help from mental health services, which includes the need, trust, acceptance, and expectation of psychological help behavior. The Theory of Planned Behavior (TPB) developed by Ajzen (15) states that an individual’s actual behavior is indirectly influenced by behavioral intention, and three constructs predict behavioral intention: attitudes, subjective norms, and perceived behavioral control. Among them, studies (16, 17) have shown that individual attitudes are the strongest predictor of help-seeking intentions and a crucial structure in help-seeking research and practice. In addition, TPB also emphasizes the high correlation between attitude and behavior, that is, individuals will evaluate their liking or disliking of certain behaviors, and then influence their actual behaviors by influencing their behavioral intention. Many empirical studies (5, 18) have demonstrated that the mental help-seeking attitude is highly correlated with the help-seeking process. In addition, in studies where the intention was not measured, attitude also directly explained differences in actual future help-seeking behavior (19). In other words, the more positive the individual’s attitude towards mental help-seeking, the more likely it is to take help-seeking behavior into practice. Therefore, a positive mental help-seeking attitude is the first step towards promoting help-seeking behavior, which can facilitate access to mental health and wellness services. However, the previous systematic reviews (20–23) of mental help-seeking mostly focused on help-seeking behavior, which is a comprehensive process, including the promotion and hindrance factors, and there are few studies on mental help-seeking attitudes. Given the critical role of mental help-seeking attitudes in help-seeking research and practice, it is important to be able to effectively and accurately assess this structure via patient-reported outcome measures (PROMs). At present, many measuring tools can be used to evaluate the attitude of psychological help, with different contents and evaluation methods, and the quality is uneven. Simultaneously, we found no study that systematically evaluated and compared measures of mental help-seeking attitudes to help clinicians and researchers select appropriate scales for specific uses.

The Consensus-based Standards for the Selection of Health Measurement Instruments (COSMIN) (24) methodology evaluates the measurement tools from the quality of measurement properties and methodological quality, and comprehensively assesses the evidence quality of each measurement property, thus forming the final recommendation of the measurement tools. Therefore, the COSMIN methodology facilitates a systematic review of measurement instruments. This study comprehensively and systematically reviewed the mental help-seeking attitude assessment tools according to the COSMIN guidelines to provide reference to researchers on the selection, application, and development of related tools.

## 2. Methods

### 2.1. Design

This systematic review was carried out following the COSMIN (24) guideline. The Preferred Reporting Items for Systematic reviews and Meta-Analyses (PRISMA) (25) guideline was used in reporting the study and was registered with PROSPERO (CRD42022382992).

### 2.2. Search strategy

Studies on mental help-seeking attitudes were comprehensively searched in PubMed, Embase, Web of Science, Medline, APA, CINAHL, Sinomed, CNKI, and WanFang since the establishment of the database until November 30, 2022, and the language of the study is not limited. To ensure the inclusion of all available and relevant preliminary studies, this study used Medical Subject Headings (MeSH) terms and free-text words to identify studies concerning the measured constructs. According to COSMIN guidelines and the advice of relevant experts, two authors developed a search strategy. Key terms were applied to each database: mental help-seeking attitude OR psychological help-seeking attitude OR attitudes toward seeking professional psychological help OR attitudes toward seeking mental help OR Psychological help attitude OR help-seeking attitude. Supplementary document 1 provides a detailed search strategy in the PubMed database as an example. In addition, we manually searched the reference lists of relevant articles as other sources to ensure the inclusion of additional research.

### 2.3. Eligibility criteria

The following inclusion criteria were applied: (1) Validation studies of measures assessing constructs relating to mental help-seeking attitudes. (2) Studies that determined at least one measurement property of PROMs. The exclusion criteria included the following: (1) the pre-experiment of measurement tools or the research of application status; (2) Secondary research (review, systematic review, etc.); (3) Repeated studies from multiple databases; (4) Studies for which the full text is not available.

### 2.4. Study selection

All available records for database searches are uploaded to EndNote (version X9, Clarivate Analytics). After the removal of duplicates, the titles and abstracts were reviewed by two researchers respectively, and studies not meeting the inclusion and exclusion criteria were removed at this stage. Any discrepancies were resolved by joint discussion or referral to the third author, and a list of the articles to be included in this review was determined. In case of non-English or Chinese published articles, literature should be processed by professional translators before the researchers review it. In addition, the reasons for excluding the study at the full-text screening stage were recorded. The process of study selection will be shown in the PRISMA flowchart.

### 2.5. Data extraction

Two researchers independently extracted data, including measurement instruments, the first author, publication year, country and language, target population, sample size, number of dimensions and number of items, scoring method, and the retest time. The main findings on measurement properties included content validity, structural validity, internal consistency, cross-cultural validity/measurement invariance, test–retest reliability, criterion validity, and hypotheses testing (24).

### 2.6. Quality appraisal

Two researchers used the COSMIN (24) guideline for systematic reviews to independently evaluate the methodological quality and the measurement property of the mental help-seeking attitude scales, and cross-checked the results. In case of disagreement, the dispute shall be settled through consultation with the third reviewer. A narrative analysis was used to summarize and analyze the measurement property and methodological quality results of the mental help-seeking attitude assessment instruments.

#### 2.6.1. Methodological quality assessment of included studies

This study used the COSMIN Risk of Bias Checklist (26) to evaluate the methodological quality of the scales, detailing an instrument’s development, content validity, construct validity, internal consistency, cross-cultural validity\measurement invariance, reliability, measurement error, criterion validity, hypotheses testing for construct validity, and responsiveness. A 4-point scoring system was used to rate the methodological quality of each study, that is “very good (V),” “adequate (A),” “doubtful (D)” or “inadequate (I).” The methodological quality score for each property was determined by taking the lowest rating of any item in each box - worst score counts principle.

#### 2.6.2. Measurement property assessment of the instruments

The quality criteria COSMIN checklist was developed by Terwee et al. (27) as a framework to evaluate the measurement property of each study. The checklist covers nine measurement properties: content validity, structural validity, internal consistency, cross-cultural validity, reliability, measurement error, criterion validity, hypothesis testing, and responsiveness, and sets the rating level as “sufficient (+),” “insufficient (−),” “indeterminate (?).” After data synthesis, when the ratings of each study are consistent, the overall rating of the measurement property is also rated as “sufficient (+)” or “insufficient (−)” or “indeterminate (?).” When the rating of each study is inconsistent, we can explore the possible reasons. If the explanation is reasonable, subgroup analyses can be performed and ratings can be provided by the subgroup. If the explanation was unreasonable, the overall rating of the measurement property was rated as “inconsistent (±).”

#### 2.6.3. Summarizing the evidence and grading the quality of the evidence

We used a modified Grading of Recommendations, Assessment, Development, and Evaluation (GRADE) (28) system to assess the overall quality of evidence. According to the risk of bias, inconsistency, indirection, and imprecision, the quality of evidence was divided into four levels: high, moderate, low, and very low.

At last, based on the objective evaluation results of the evidence, the recommendation opinions of the instruments are formed, and the intensity of the recommendation opinions is marked, which are divided into a strong recommendation (A), weak recommendation (B), and no recommendation (C). The criteria for category A are sufficient content validity (any level of evidence) and sufficient internal consistency (evidence of at least low quality). The criteria for category C are insufficient measurement property (evidence level is high). Category B is between category A and category C, and more studies are needed to verify the measurement characteristics.

## 3. Results

### 3.1. Search results

**Figure 1:**
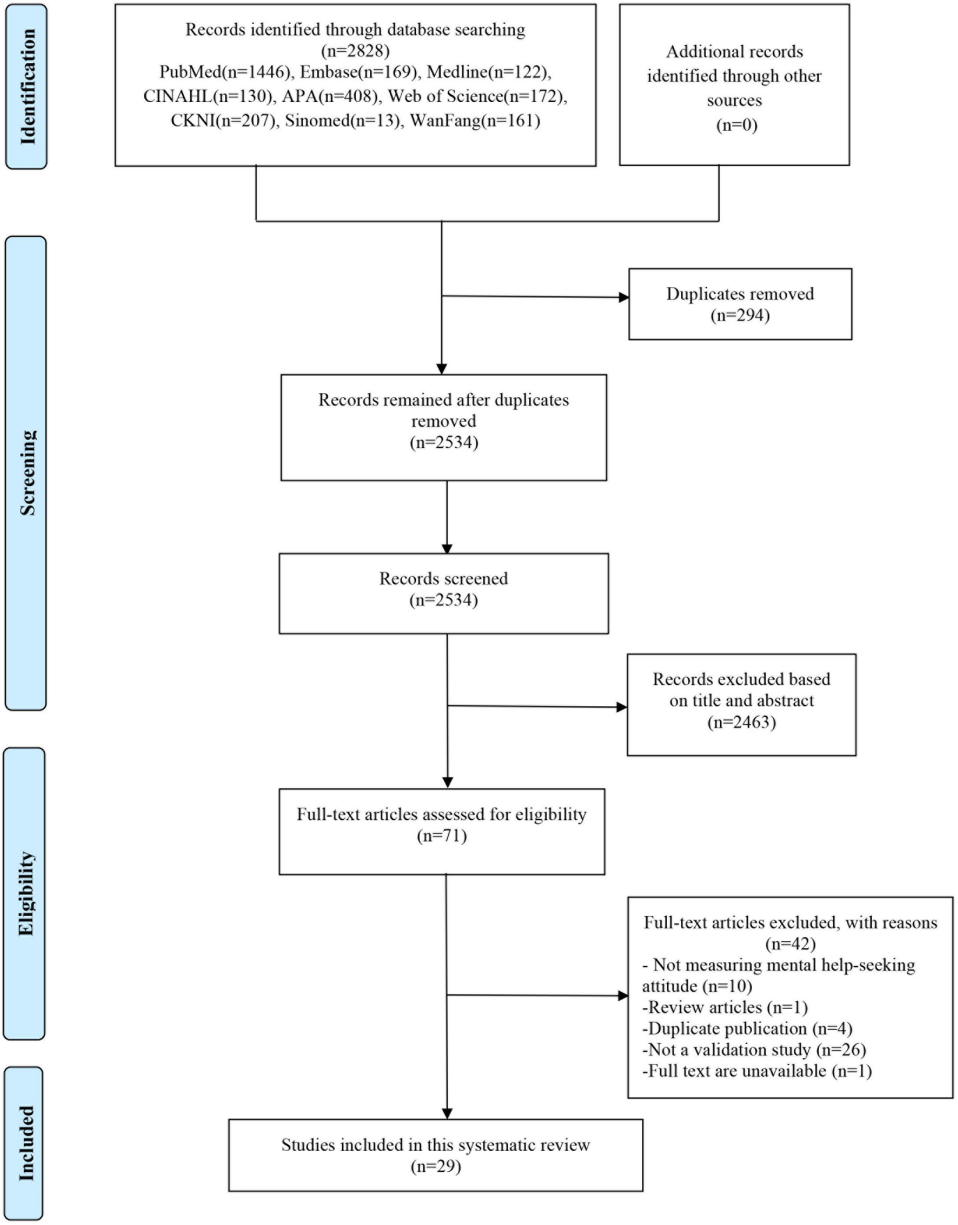
Flowchart of the identification and selection of studies.

Of the 2,828 studies retrieved from the databases, 294 duplicates were removed. Upon screening the titles and abstracts, another 2,463 studies were removed. For the remaining 71 studies, their full texts were retrieved, of which 42 were removed with reasons, leaving 29 studies (14, 29–56) in the review. A flowchart of the literature screening process is shown in Figure 1.

### 3.2. General characteristics of the instruments included

After retrieval, a total of 29 studies representing 13 instruments conducted between 1970 and 2021 were included. Among the 13 measurement instruments, Attitudes Toward Seeking Professional Psychological Help Scale-Short Form (ATSPPH-SF) is the scale that has been evaluated most frequently, with a total of 10 studies, followed by Inventory of Attitudes Toward Seeking Mental Health Services (IASMHS) (6 studies) and Attitudes Toward Seeking Professional Psychological Help Scale (ATSPPH) (3 studies), and the rest have only one study.

The number of patients in the studies included in this systematic review ranged from 150 to 3,006, with 11 studies involving college students, and the rest involving community residents, adults, future psychotherapy practitioners, parents, and immigrant adolescents. Studies were conducted in the United States (*n* = 6), China (*n* = 11), Canada (*n* = 2), Singapore (*n* = 2), the Greek (*n* = 1), Italy (*n* = 1), Jordan (*n* = 1), France (*n* = 1), Austria (*n* = 1), the Philippines (*n* = 1), Germany (*n* = 1) and Turkey (*n* = 1). The number of scale items varied from 7 to 43, and the subscales were considered to range from one to five dimensions. In addition, 10 measurement instruments had varying recall periods from 1 to 4 weeks. Table 1 shows the characteristics of the instruments included in the systematic review.

**Table 1:** Characteristics of included studies.

PROM	Authors (year)	Country (language)	Target population	Sample size	(Sub)scale(s) (number of items)	Subscales	Recall period
ATSPPH	Fischer and Turner (1970)	The USA (English)	Students	960	29 Items 5-Point Likert scale	Recognition of need for psychological help Stigma tolerance Confidence in mental health professionals Interpersonal openness	2w
	Hao and Liang (2007)	China (Chinese)	College students	620	29 Items 5-Point Likert scale	Recognition of need for psychological help Stigma tolerance Confidence in mental health professionals Interpersonal openness	NR
	Xu (2007)	China (Chinese)	College students	150	29 Items 5-Point Likert scale	Recognition of need for psychological help Stigma tolerance Confidence in mental health professionals Interpersonal openness	NR
ATSPPH-SF	Ang et al. (2007)	Singapore (English)	College students, teachers	331	9 Items 4-Point Likert scale	Unidimensional scale	NR
	Elhai et al. (2008)	The USA (English)	College students	296	7 Items 4-Point Likert scale	Openness to seeking treatment for emotional problems Value and need in seeking treatment	NR
	Fang et al. (2011)	China (Chinese)	Chinese	338	7 Items 4-Point Likert scale	Confidence in the value of counseling Motivation for seeking counseling	NR
	Picco et al. (2016)	Singapore (English)	Adults	3,006	10 Items 4-Point Likert scale	Openness to seeking professional help Value in seeking professional help Preference to cope on one’s own	NR
	Kong and Hao (2018)	China (Chinese)	Adults	1,186	10 Items 4-Point Likert scale	Openness to seeking professional help Value in seeking professional help Preference to cope on one’s own	2w
	Fang et al. (2019)	China (Chinese)	Community residents	1720	10 Items 4-Point Likert scale	Openness to seeking treatment for emotional problems Value and need in seeking treatment	2w
	Efstathiou et al. (2019)	The Greek (Greek)	College students	1,381	10 Items 4-Point Likert scale	Openness to seeking treatment for emotional problems Value and need in seeking treatment	4w
	Rossi and Mannarini (2019)	Italy (Italian)	College students	320	10 Items 4-Point Likert scale	Openness to seeking treatment for emotional problems Value and need in seeking treatment	NR
	Rayan et al. (2020)	Jordan (Arabic)	College students	519	10 Items 4-Point Likert scale	Unidimensional scale	NR
	Torres et al. (2021)	The USA (Spanish)	Latino adult individuals	437	10 Items 4-Point Likert scale	Openness to seeking treatment for emotional problems Value and need in seeking treatment	NR
IASMHS	Mackenzie et al. (2004)	Canada (English)	College students	293	24 items 5-point Likert scale	Psychological openness Help-seeking propensity Indifference to stigma	3w
	Lheureux (2015)	France (French)	Adults	702	24 Items 5-Point Likert scale	Psychological openness Help-seeking propensity Indifference to stigma	NR
	Kantor et al. (2017)	Austria (English)	Adult survivors of institutional abuse	220	24 Items 5-Point Likert scale	Psychological openness Help-seeking propensity Indifference to stigma	NR
	Tieu et al. (2018)	Canada (Chinese)	Chinese-Canadian elders	200	20 Items 5-Point Likert scale	Psychological openness Help-seeking propensity Indifference to stigma	NR
	Tuliao et al. (2019)	The Philippines (Filipino)	The Philippines	733	20 Items 5-Point Likert scale	Psychological openness Help-seeking propensity Indifference to stigma	NR
	Zhou et al. (2019)	Germany (German/Chinese)	College students	829	24 Items 5-Point Likert scale	Psychological openness Help-seeking propensity Indifference to stigma	NR
TATSPS	Farber et al. (2000)	The USA (English)	Future psychotherapy practitioners	275	26 Items 5-Point Likert scale	Importance for professional growth/effectiveness Concern with professional credibility Concern with confidentiality Need for self-sufficiency	NR
ATSPPHQ-M	Cui (2003)	China (Chinese)	Middle school students	346	20 Items 5-Point Likert scale	Trust in Psychological Professionals Acceptance of help-seeking behaviour Evaluation of psychological help Expectations of the effectiveness of the help Tendency to ask for help	3w
ASPH	Türküm et al. (2004)	Turkey (English)	College students	356	43 Items 5-Point Likert scale	Confidence for getting psychological help Beliefs about the functions of psychological help Endurance against labelling Self-disclosure	15 Days
ATSPPHQ-C_1_	Yang (2006)	China (Chinese)	College students	400	15 Items 5-Point Likert scale	Confidence in mental health professionals Recognition of need for professional psychological help Individual’s tolerance to social stereotypes Openness to asking for help	4w
ATSPPHQ-C_2_	Wei (2008)	China (Chinese)	College students	500	20 items 5-point Likert scale	Recognition of need for psychological help Expectations of the effectiveness of the help Confidence in psychological professional services Acceptance of asking for help	NR
ATSPPHQ-CS	Li (2010)	China (Chinese)	Civil servants	200	21 Items 5-Point Likert scale	Need awareness Stereotype tolerance Trust in experts Interpersonal openness	NR
PATPSI	Turner (2012)	The USA (English)	Parents	250	26 Items 6-Point Likert scale	Help-seeking attitudes Help-seeking intentions Stigmatization	1w
ATSPPH-PP	Pan (2014)	China (Chinese)	Pupil’s parents	360	24 Items 5-Point Likert scale	Evaluation Doubts Problem identification Tendency to ask for help	NR
IAPAQ	Jian (2018)	China (Chinese)	Immigrant adolescents	330	20 Items 5-Point Likert scale	Evaluation Acceptance Willingness and inclination Expectations of the effectiveness Trust of consultants	NR
MHSAS	Hammer et al. (2018)	The USA (English)	Community adults	857	9 Items 7-Point Likert scale	Unidimensional scale	3w

ATSPPH, Attitudes Toward Seeking Professional Psychological Help Scale; ATSPPH-SF, Attitudes Toward Seeking Professional Psychological Help Scale-Short Form; IASMHS, Inventory of Attitudes Toward Seeking Mental Health Services; TATSPS, Trainees’ Attitudes Toward Seeking Psychotherapy Scale; ATSPPHQ-M, Attitude Toward Seeking Professional Psychological Help Questionnaire-Middle School Students; ASPH, Attitudes Toward Seeking Psychological Help Scale; ATSPPHQ-C1, Attitude Toward Seeking Professional Psychological Help Questionnaire-College Students 1; ATSPPHQ-C_2_, Attitude Toward Seeking Professional Psychological Help Questionnaire-College Students 2; ATSPPHQ-CS, Attitude Toward Seeking Professional Psychological Help Questionnaire-Civil Servants; PATPSI, Parental Attitudes Toward Psychological Services Inventory; ATSPPHQ-PP, Attitude Toward Seeking Professional Psychological Help Questionnaire-Pupil’s Parents; IAIPQ, Immigrated Adolescents’ Psychological Attitude Questionnaire; MHSAS, Mental Help Seeking Attitudes Scale; PROM, patient-reported outcome measure; NR, not reported.

### 3.3. Methodological quality of the included studies and measurement property assessment of the instruments

Data obtained in the measurement properties assessment of the selected instruments and the methodological quality results were summarized in Table 2. This systematic review evaluated the content validity (*n* = 16), structural validity (*n* = 29), internal consistency (*n* = 28), cross-cultural validity/measurement invariance (*n* = 7), test–retest reliability (*n* = 11), criterion validity (*n* = 4), and hypothesis testing (*n* = 1) of the included instrument.

**Table 2:** Methodological quality of the included studies and measurement property assessment of the instruments.

PROM	Reference	Content validity			Structural validity		Internal consistency		Cross-cultural validity\Measurement invariance		Reliability		Criterion validity		Hypothesis testing	
		RE	CV	CB	Index	M/P	Cronbach’s α	M/P	Index	M/P	Index	M/P	Index	M/P	Index	M/P
ATSPPH	Fischer et al. (36)	D^a,b^/?	D^a,b^/?	D^b^/?	EFA: 4 factors	A/?	0.62–0.74	V/−			*r* = 0.89	D/?				
	Hao and Liang (37)	D^a,b^/?	D^a,b^/?	D^b^/?	CFA: 4 factors CFI = 0.87	V/−	0.562–0.662	V/−								
	Xu (54)				CFA: 4 factors CFI = 0.92	A/−	0.568–0.759	V/−								
ATSPPH-SF	Ang et al. (29)				CFI = 0.982 (College students) CFI = 0.979 (Teachers)	V/+	0.71 (college student), 0.70 (teacher)	V/+	MGCFA	D/+						
	Elhai et al. (32)				CFA&EFA: 2 factors CFI = 0.94	V/−	0.77	I/−	MGCFA	D/+						
	Fang et al. (33)				CFA&EFA: 2 factors	V/?	0.48–0.59	V/−								
	Picco et al. (45)			D^b^/?	CFA&EFA: 3 factors CFI = 0.978	V/+										
	Kong and Hao (40)				CFA&EFA: 3 factors CFI = 0.98	V/+	0.71–0.75	V/+			ICC = 0.82	D/+	*r* = 0.67	V/−		
	Fang et al. (34)	D^a,b^/?	D^a,b^/?	D^b^/?	CFA&EFA: 2 factors RMSEA = 0.054	V/+	0.657–0.741	V/−			ICC = 0.895	D/+				
	Efstathiou et al. (31)				CFA&EFA: 2 factors CFI = 0.969	V/+	0.64–0.70	V/−			ICC = 0.89	D/+				
	Rossi and Mannarini (47)				CFA: 2 factors CFI = 0.993	V/+	0.753–0.895	V/+	MGCFA	D/+						
	Rayan et al. (46)			D^b^/?	CFA&EFA: 1 factor RMSEA = 0.05	V/+	0.72	V/+					*r* = 0.85	V/+		
	Torres et al. (49)				CFA&EFA: 2 factors CFI = 0.962	V/+	0.70	I/−	MGCFA	D/−						
IASMHS	Mackenzie et al. (43)	D^b^/?	D^b^/?	D^b^/?	CFA&EFA: 3 factors RMSEA = 0.04	V/+	0.76–0.82	V/+			*r* = 0.85	D/?				
	Lheureux (41)				CFA&EFA: 3 factors CFI = 0.96	V/+	0.71–0.76	V/+								
	Kantor et al. (39)				CFA&EFA: 3 factors RMSEA = 0.059	V/+	0.67–0.80	V/−								
	Tieu et al. (48)				CFA&EFA: 3 factors	V/?	0.59–0.79	V/−								
	Tuliao et al. (50)				RMSEA = 0.047 (College student) RMSEA = 0.036 (Workers)	V/+	0.656–0.842 (College student) 0.582–0.757 (Workers)	V/−	MGCFA	D/−						
	Zhou et al. (56)				RMSEA = 0.054 (China) RMSEA = 0.063 (Germany)	V/+ (China) V/− (Germany)	0.62–0.80 (China) 0.70–0.85 (Germany)	V/− (China) V/+ (Germany)	MGCFA	D/−						
TATSPS	Farber et al. (35)	D^b^/?	D^b^/?	D^b^/?	EFA: 4 factors	A/?	0.71–0.87	V/+								
ATSPPHQ-M	Cui (30)	D^b^/?	D^b^/?	D^b^/?	EFA: 5 factors	A/?	0.84	I/−			ICC = 0.78	D/+				
ASPH	Türküm et al. (51)	D^a,b^/?	D^a,b^/?	D^b^/?	EFA: 4 factors	A/?	0.68–0.76	V/−			*r* = 0.99	D/?				
ATSPPHQ-C_1_	Yang (55)	D^a/^?	D^a^/?		CFA&EFA: 4 factors CFI = 0.98	V/+	0.7164–0.8369	V/+			ICC = 0.74	D/+				
ATSPPHQ-C_2_	Wei (53)	D^a,b^/?	D^a,b^/?		CFA&EFA: 4 factors RMSEA = 0.056	V/+	0.562–0.676	V/−								
ATSPPHQ-CS	Li (42)	D^a,b^/?	D^a,b^/?	D^b^/?	CFA&EFA: 4 factors CFI = 0.93	V/−	0.72–0.83	V/+								
PATPSI	Turner (52)	D^a,b^/?	D^a,b^/?	D^b^/?	CFA: 3 factors RMSEA = 0.051	V/+	0.72–0.92	V/+			*r* = 0.67	D/?				
ATSPPHQ-PP	Pan (44)	D^a^/?	D^a^/?		CFA and EFA: 4 factors RMSEA = 0.052	V/+	0.735–0.813	V/+								
IAPAQ	Jian (38)	D^a,b^/?	D^a,b^/?	D^b^/?	CFA and EFA: 5 factors CFI = 0.903	V/−	0.625–0.709	V/−								
MHSAS	Hammer et al. (41)	A^a^, D^b^/+	A^a^,D^b^/+		CFA and EFA: 1 factors CFI = 0.96	V/+	0.93	V/+	MGCFA	D/+	ICC = 0.86	D/+			2 Comparison instruments/2 Comparison subgroups	A/+

Empty cells indicate that no information was available on this item. Measurement error and responsiveness were not determined in the studies. RE, relevance; CV, comprehensiveness; CB, comprehensibility; M/P, methodology/property; V, very good; A, adequate; D, doubtful; I, inadequate; ^a^Professional consultation results; ^b^Patient consultation results; (+), sufficient; (−), insufficient; (?), indeterminate; CFA, confirmatory factor analysis; EFA, exploratory factor analysis; MGCFA, multi-group confirmatory factor analysis; CFI, comparative fit index; RMSEA, root mean square error of approximation; ICC, intraclass correlation coefficient; r, Pearson product–moment correlation coefficient; NR, not reported.

#### 3.3.1. Content validity

Content validity refers to the degree of agreement between the content of PROMs and the measured construct, which is considered to be the most important measurement characteristic of PROM (57). According to the COSMIN guidelines (24), content validity is described in aspects of relevance, comprehensiveness, and comprehensibility.

Of the 16 studies (14, 30, 34–38, 42–46, 51–53, 55) that assessed content validity, 5 (30, 35, 43, 45, 46) only asked the target population about their perceptions of scale items, 2 (44, 55) assessed the content validity of the scale through expert consultation, and 9 (14, 34, 36–38, 42, 51–53) asked both the target population and experts. Of these, two studies (45, 46) evaluated comprehensibility only, and four(14, 44, 53, 55)evaluated relevance and comprehensiveness only.

In the 6 studies (37, 38, 44, 45, 51, 53), qualitative interviews were used to investigate the target population, but the interview guide and content were not specified, and the data analysis methods were not clear, so the methodological quality of the research was “doubtful.” The study of Hammer et al. (14) used a questionnaire survey to evaluate the relevance, comprehensiveness, and comprehensibility of the scale items among community adults, and the analysis approach was appropriate but not clearly described, so the methodological quality was “adequate.” In addition, since only 5 professionals from relevant disciplines were quantitatively surveyed in the study of Hammer et al., the methodological quality regarding the relevance and comprehensiveness of professionals is “doubtful.” Other studies only used quantitative survey methods to evaluate content validity, and the description of the research process/statistical methods was not clear, so the methodological quality of these studies is “doubtful.”

#### 3.3.2. Structural validity

Structural validity refers to the degree to which the score of a PROM adequately reflects the dimensions of the construct to be measured (58). All included studies were evaluated for structural validity. In terms of methodological quality, was in the 4 studies (30, 35, 36, 43) conducted only Exploratory Factor Analysis (EFA), so we rated them as adequate for structural validity. One study (54) performed confirmatory factor analysis (CFA), but the structural validity of this study was also rated as adequate because there were only 150 subjects, less than 7 times the number of items. CFA was performed in the remaining 24 studies, and the sample size was sufficient, so the structural validity was rated as very good. In terms of instrumental measurement property assessment, 17 (14, 29, 31, 34, 39–47, 49, 50, 52, 55) Of the 24 studies reported the Comparative fit Index (CFI) > 0.95, or Root Mean Square Error of Approximation (RMSEA) < 0.06, so the structural validity of these studies was adequate.

#### 3.3.3. Internal consistency

Internal consistency, defined as the degree of interrelatedness among the items, is usually assessed by Cronbach’s alpha (58). Twenty-eight studies measured the internal consistency, 3 (30, 32, 49) of which were rated as “inadequate” for not giving Cronbach’s alpha for the subscale. Other studies reported the Cronbach’s alpha of all dimensions, and the methodological quality was “very good.” 15 studies (30–34, 36–39, 48–51, 53, 54) reported Cronbach alpha below 0.70, so the internal consistency was rated as “insufficient.”

#### 3.3.4. Cross-cultural validity/measurement invariance

Cross-cultural validity**/**measurement invariance refers to the degree of consistency in the score of PROMs items when measured across different cultural groups (59). A total of 7 studies (14, 29, 32, 47, 49, 50, 56) used multi-group CFA (MGCFA) to verify the measurement variability of the instruments in different cultural groups, however, they did not specify whether other relevant characteristics except for the group variable were similar, so the methodological quality of the research was “doubtful.” 3 studies (49, 50, 56) found differences in the grouping variables, so the cross-cultural validity/measurement invariance was “insufficient.”

#### 3.3.5. Reliability

Reliability is defined as the extent to which a measurement is free of measurement error and can be tested with repeated measurements in stable patients at appropriate intervals under similar test conditions (60). Ten studies (14, 30, 31, 34, 36, 40, 43, 51, 52, 55) evaluated the reliability, but the methodology quality was “doubtful” because it failed to detail whether the characteristics of the test subjects, the test conditions, and the constructs to be measured were stable during the interval, and failed to provide the basis for the time interval. For six studies (14, 30, 31, 34, 40, 55), the intraclass correlation coefficient (ICC) was calculated, and ICC > 0.70, so the reliability was rated as “sufficient.” Other studies only calculated the Pearson correlation coefficient, so reliability was rated as “indeterminate.”

#### 3.3.6. Criterion validity

Criterion validity refers to the degree to which the scores of a PROM are an adequate reflection of a gold standard. Two studies (40, 46) assessed the criterion validity, all of which took ATSPPH as the gold standard and calculated the correlation, so the methodology quality was rated as “very good.” Only one study (46) calculated a correlation greater than 0.70 and was therefore rated “sufficient.”

#### 3.3.7. Hypothesis testing

In the assessment of hypothesis testing, there are two parts, a and b. Part a is the comparison with other outcome measurement instruments, and part b is the comparison between subgroups. In the studies, part a or part b or both can be evaluated, depending on the type of comparison. In this systematic review, only one study evaluated the hypothesis testing, Part a is the comparison with ATSPPH and IASMH, and Part b is the comparison between subgroups of men and women, and between subgroups of whether they had previously sought mental health services. However, Mental Help Seeking Attitudes Scales (MHSAS) only gave the correlation coefficient instead of the mean and standard deviation, so the methodology quality was reduced from “very good” to “adequate.” The research results of Hammer et al. were consistent with the hypothesis, so they were rated as “sufficient.”

### 3.4. Summary of evidence and grading of the quality of evidence

This section summarizes the overall ratings and quality of evidence for all assessment tools, and Table 3 was formed. A total of 13 measurement tools were included in our study, but none of them measured measurement error and responsiveness.

**Table 3:** Summary of evidence and grading of the quality of evidence.

PROM	Content validity		Structural validity		Internal consistency		Cross-cultural validity\Measurement invariance		Reliability		Criterion validity		Hypothesis testing		Level of recommendation
	S	QE	S	QE	S	QE	S	QE	S	QE	S	QE	S	QE	
ATSPPH	?	M	−	M	−	H			?	L					C
ATSPPH-SF	?	M	+	M	±	L	+	M	+	M	±	M			B
IASMHS	?	M	+	M	±	M	−	M	?	L					B
TATSPS	?	M	?	M	+	H					−	H			C
ATSPPHQ-M	?	M	?	M	−	L			+	L					B
ASPH	?	M	?	M	−	H			?	L					C
ATSPPHQ-C_1_	?	M	+	H	+	H			+	H					B
ATSPPHQ-C_2_	?	M	+	H	−	H									C
ATSPPHQ-CS	?	M	−	H	+	H									C
PATPSI	?	M	+	H	+	H			?	L					B
ATSPPHQ-PP	?	M	+	H	+	H									B
IAPAQ	?	M	−	H	−	H									C
MHSAS	+	M	+	H	+	H	+	L	+	L			+	H	A

Empty cells indicate that no information was available on this item. S, summarized results; (+), sufficient; (−), insufficient; (?), indeterminate; (±), inconsistent; QE, quality of evidence; (H), high; (M), moderate; (L), low; (VL), very low; (A), strong recommendation; (B), weak recommendation; (C), no recommendation.

In terms of measurement property quality, all 13 tools involved the assessment of content validity, but only the study of Hammer et al. was rated as “sufficient,” and the rest tools were “indeterminate.” Among the structural validity evaluation tools, the structural validity of ATSPPH, Attitude Toward Seeking Professional Psychological Help Questionnaire-Civil Servants (ATSPPHQ-CS), and Immigrated Adolescents’ Psychological Attitude Questionnaire (IAPAQ) was rated as “insufficient,” the structural validity of Trainees’ Attitudes Toward Seeking Psychotherapy Scale (TATSPS), Attitude Toward Seeking Professional Psychological Help Questionnaire-Middle School Students (ATSPPHQ-M), and Attitudes Toward Seeking Psychological Help Scale (ASPH) was rated as “indeterminate,” and the structural validity of other evaluation tools was “sufficient.” Internal consistency was evaluated for 13 tools, with six rated as “sufficient” and five rated as “insufficient.” In addition, the internal consistency of ATSPPH-SF and IASMHS is “inconsistent.” Cross-cultural validity/measurement invariance was evaluated by three assessment tools, of which ATSPPH-SF and MHSAS were rated as “sufficient.” 8 assessment tools were used to evaluate reliability, ATSPPH-SF, ATSPPHQ-M, Attitude Toward Seeking Professional Psychological Help Questionnaire-College Students 1 (ATSPPHQ-C_1_), and MHSAS were rated as “sufficient,” while the rest were rated as “indeterminate.” Only 2 assessment tools evaluated the criterion validity, among which ATSPPH-SF was “inconsistent” and TATSPS was “insufficient.” MHSAS measured the hypothesis test and was rated “sufficient.”

The quality of evidence is affected by four downgrading factors: risk of bias, inconsistency, indirectness, and imprecision. In this study, due to the impact of bias risk and inconsistency, the overall quality of each measurement property is mostly moderate or low, especially content validity, structural validity, cross-cultural validity/measurement invariance, and reliability. Based on the COSMIN, measurement instruments can be classified as strongly recommended (A), weakly recommended (B), and not recommended (C). Six of the 13 measurement instruments were rated as B, 6 were rated as C, and only MHSAS was rated as A.

## 4. Discussion

This systematic review is the first psychometric review concerning the measurement properties of mental help-seeking attitude instruments based on the COSMIN checklist. All stages of this study are conducted according to PRISMA and COSMIN guidelines, which have a high evidence level. Through the comprehensive and systematic evaluation of the included research, it found that the attitude of mental help-seekers mainly focuses on the cognition of mental health, overall evaluation of psychological help, tolerance of stigma, interpersonal openness, need for self-sufficiency, and the trust of psychological professionals. However, different instruments have obvious differences in the quality of measurement properties, and the quality of the instruments is uneven. Therefore, the problems found in this study were summarized to provide a reference for the development and verification of a high-quality psychological help-seeking attitude scale in the future.

In assessing the methodological quality of studies, content validity is the most important measurement property of a PROM (27). Through the evaluation process of PROM development, this study found that most of the included instruments were compiled according to the results of literature review, expert consultation, target population interview, or questionnaire survey. ASPH, Attitude Toward Seeking Professional Psychological Help Questionnaire-College Students 2 (ATSPPHQ-C_2_), Attitude Toward Seeking Professional Psychological Help Questionnaire-Pupil’s Parents (ATSPPHQ-PP), and IAPAQ conducted qualitative interviews with the target population but did not describe whether there was an interview guide, skilled interviewers, and a specific interview process. It is also unclear about interview content saturation and qualitative data analysis process, so the quality of content validity methodology is “doubtful.” The rest of the assessment instruments only conduct questionnaire surveys on the target population, among which only the content validity of MHSAS has “adequate” methodological quality, and the data analysis process of other instruments was not clear, so the methodological quality was “doubtful.” This resulted in low recommendation ratings for most measurement instruments. Therefore, the general problem of content validity research is the lack of qualitative interviews with the target population and detailed and standardized descriptions of research methods. COSMIN recommends the use of qualitative research to investigate the relevance, comprehensiveness, and comprehensibility of items from patients or professionals to measure content validity. Future studies can refer to the COSMIN checklist (24) to improve the PROM design, and use in-depth individual interviews or focus group interviews to understand the views of research subjects on the content of PROM so that the research subjects can participate in the verification and evaluation process. In addition, the process of data analysis should be described in detail, especially the process of qualitative research data analysis.

In this review, the included studies were all based on classical test theory (CTT), and factor analysis is the preferred method for evaluating structural validity in CTT (61). Factor analysis is a broad term that refers to a set of statistical methods for extracting common regression coefficient from a number of observed variables (62). There are two main approaches: exploratory factor analysis (EFA) and CFA. CFA applies to the situation where the dimension of the measured construct is determined, while EFA applies to the situation where the dimension of the measured construct is uncertain. The fundamental difference between the two is whether there is a prior theory or knowledge. Compared with EFA, CFA can describe the relationship between measurement items and factors, and directly verify this relationship or model (63). Many of the included studies only conducted EFA to produce a theory of internal structure, but not CFA, so it was impossible to evaluate the degree of conformity between the factor structure and the sample data defined by the theory. In fact, CFA and EFA are two stages of the research process, and only the combination of the two can make the research more in-depth. Therefore, for PROMs lacking a theoretical basis, when evaluating the structural validity, it is recommended to first use EFA to clarify its internal structure, and then apply CFA to analyze the relationship between measurement items and factors.

Cross-cultural validity/measurement invariance was evaluated in ATSPPH-SF, IASMHS, and MHSAS, but the quality of evidence was not high. An ideal measurement instrument should have a stable structure when measured in different cultural groups. Therefore, when the instrument is used in different cultural groups, attention should be paid to the measurement invariance between groups and whether there is differential item functioning (DIF) of scale items. Only MHSAS evaluated the hypothesis testing of structural validity. Hypothesis testing for structural validity refers to the extent to which scale scores are consistent with the hypothesis. The more specific the hypothesis and the more hypotheses tested, the more evidence supporting structural validity. Further research needs to focus more on this measurement property. In terms of criterion validity, there is no gold standard in the field of psychological help-seeking attitudes. However, the COSMIN guidelines state that the original scale can serve as the “gold standard” for the newly developed shorter version. Therefore, researchers can compare the short version scale with the original scale to verify whether it has good criterion validity.

All 13 assessment instruments evaluated internal consistency, there were 3 studies only giving the Cronbach alpha value of the total scale, so the methodology quality of internal consistency was rated as “inadequate.” The COSMIN guidelines state that the internal consistency of each subscale should be calculated when the scale presents multidimensional dimensions. In future development and verification, the Cronbach alpha of each subscale should be given on the premise of clear structural validity. In the scales assessing reliability, the quality of evidence was mostly low, because these scales did not address the reason for the choice of the measurement interval, nor did they indicate whether subjects were stable in the interim period on the construct to be measured. In addition, COSMIN points out that when evaluating reliability, the preferred choice for continuous scores is ICC, for dichotomous/nominal/ordinal scores is Kappa, and for ordered scores is the weighted Kappa. Besides, the ICC model is a two-way random effects model, which takes into account both the variation within the subject and the systemic variation, while the Person or Spearman correlation coefficient does not consider systematic variation. All the included studies were continuous scores, but 4 studies performed only the Person correlation coefficient. Measurement error and responsiveness were not reported for any of the 13 instruments in this study. The measurement error refers to the systematic and random errors of the measured scores, including the Standard Error of Mean (SEM) and the Minimal Detectable Change (MDC). Responsiveness refers to the ability of the scale to examine score changes over time. At present, the psychological help attitude tools are in the development stage, and the measurement error and responsiveness can be tested to improve the scientific nature of the assessment tools.

In addition to evaluating its methodology and measuring property quality, the application population is also a key concern in the selection of assessment tools. The application population of the scales should be consistent with the characteristics of the population included in the development of the scales. However, there are differences between the original target population and the application population of most psychological help-seeking attitude assessment tools, such as ATSPPH-SF and IASMHS. Taking IASMHS as an example, Mackenzie et al. developed the scale based on college students and then applied it to different groups such as adults, Chinese-Canadian elders, and adult abuse survivors. Although the IASMHS is rated as class B based on the modified GRADE system, indicating that the IASMHS has the potential to be applied in these populations, whether the IASMHS is the best assessment tool for these specific populations needs further research to be verified. In addition, there are many targeted assessment tools developed for special groups in this study, such as TATSPS, ATSPPHQ-M, and so on. Therefore, more consideration can be given to developing targeted assessment tools in future research.

The results of this study show that MHSAS is the class A scale, ATSPPH-SF, IASMHS, ATSPPHQ-M, ATSPPHQ-C_1_, Parental Attitudes Toward Psychological Services Inventory (PATPSI), ATSPPHQ-PP is the class B scale. ATSPPH, TATSPS, ASPH, ATSPPHQ-C_2_, ATSPPHQ-CS and IAPAQ are Class C scales. Among them, the class A scale is recommended in the application population, the class B scale has application potential, but it needs further verification, and the class C scale is not recommended (28). In other words, when choosing mental help-seeking attitudes assessment tools, ATSPPH, ASPH, and ATSPPHQ-C_2_ are not recommended among college students, TATSPS is not recommended among future psychotherapy practitioners, ATSPPHQ-CS is not recommended among civil servants, and IAPAQ is not recommended among immigrant adolescents. ATSPPH-SF, IASMHS, ATSPPHQ-M, ATSPPHQ-C_1_, PATPSI, and ATSPPHQ-PP have the potential to be applied in the corresponding population. As the only Type A scale, MHSAS is recommended for community adults. MHSAS is developed based on the defects of previous assessment tools, so the scale development process is more standardized. MHSAS is a single-dimensional scale with 9 items and is based on the Theory of Planned Behavior (TPB). The items are simple and the evaluation is comprehensive. Compared with other PROMs, MHSAS has moderate-quality evidence to support its content validity and high-quality evidence to support its internal consistency. It has good clinical feasibility and can be used in future research on psychological help-seeking attitudes. However, at present, there are few validation studies on MHSAS, only one study was included, and only its recommended rating was obtained in community adults, without adaptation studies in different age groups and other populations, so the promotion degree of MHSAS in other groups is also uncertain. Follow-up studies may also explore the use of MHSAS in other populations to provide a more sufficient research basis. ATSPPH-SF was the most frequently evaluated scale, but in terms of tool development, it lacked standardization, and the research on content validity was not perfect, so it is only rated as “indeterminate” (moderate evidence). These problems also exist on other class B scales. In addition, future studies can further explore the specific mental help-seeking attitudes assessment tools of a certain special population.

### 4.1. Limitations

Overall, there are also some limitations in this study. First, as with all systematic reviews, this study has the possibility of publication bias. In addition to ATSPPH, ATSPPH-SF, and IASMHS, there is only one study report of other PROMs, and validation studies with negative results may never have been published. Second, some studies may have been conducted correctly but not described in sufficient detail according to COSMIN standards, affecting their quality ratings. Third, we included only studies that were designed to assess the measurement properties of PROMs for mental help-seeking attitudes. Finally, the target population of existing PROMs is mostly college students, and there are few studies in other groups.

## 5. Conclusion

This systematic review provides a comprehensive overview of the quality of measurement properties and methodological quality of mental help-seeking attitude instruments. The results of this review can contribute to the selection of the appropriate measuring instruments to assess psychological help-seeking attitudes. Currently, a number of PROMs of psychological help-seeking attitudes are available for use, but without evidence of an adequate development process. We categorized as A only the MHSAS which has sufficient psychometric evidence to be recommended as the most appropriate tool. ATSPPH-SF, IASMHS, ATSPPHQ-M, ATSPPHQ-C1, PATPSI, and ATSPPHQ-PP were categorized as B with the potential to be recommended and should be evaluated with further studies. Future studies should pay attention to the measurement characteristics of content validity, verify structural validity before measuring internal consistency, and fully describe the measurement characteristics of Cross-cultural validity/measurement invariance, measurement error, criterion validity, and responsiveness.

## Author contributions

CB and Y-HZ designed the study. CB, S-YC, W-WZ, and YC organized the data collection and extraction. CB, S-YC, S-RY, and R-XW performed the quality appraisal. CB and Y-HZ were responsible for drafting and critical revisions of the manuscript. All authors contributed to the article and approved the submitted version.

## Funding

This work was supported by grants from Jiangsu Province Hospital Association (grant number JSYGY-2-2021-411).

## Conflict of interest

The authors declare that the research was conducted in the absence of any commercial or financial relationships that could be construed as a potential conflict of interest.

## Publisher’s note

All claims expressed in this article are solely those of the authors and do not necessarily represent those of their affiliated organizations, or those of the publisher, the editors and the reviewers. Any product that may be evaluated in this article, or claim that may be made by its manufacturer, is not guaranteed or endorsed by the publisher.
